# Innovative Pedagogy and Design-Based Research on Flipped Learning in Higher Education

**DOI:** 10.3389/fpsyg.2021.577002

**Published:** 2021-02-18

**Authors:** Li Zhao, Wei He, Yu-Sheng Su

**Affiliations:** ^1^School of Education Science, Nanjing Normal University, Nanjing, China; ^2^Department of Computer Science and Engineering, National Taiwan Ocean University, Keelung, Taiwan

**Keywords:** flipped learning, innovative pedagogy, higher education, playful learning environment, design based research

## Abstract

In order for higher education to provide students with up-to-date knowledge and relevant skillsets for their continued learning, it needs to keep pace with innovative pedagogy and cognitive sciences to ensure inclusive and equitable quality education for all. An adequate implementation of flipped learning, which can offer undergraduates education that is appropriate in a knowledge-based society, requires moving from traditional educational models to innovative pedagogy integrated with a playful learning environment (PLE) supported by information and communications technologies (ICTs). In this paper, based on the design-based research, a task-driven instructional approach in the flipped classroom (TDIAFC) was designed and implemented for two groups of participants in an undergraduate hands-on making course in a PLE. One group consisting of 81 students as the experimental group (EG) received flipped learning instruction, and another group of 79 students as the control group (CG) received lecture-centered instruction. The EG students experienced a three-round study, with results from the first round informing the customized design of the second round and the second round informing the third round. The experimental results demonstrated that students in the EG got higher scores of summative tests and final scores than those in the CG. In particular, students’ learning performance in three domains (i.e., cognitive, affective, and psychomotor) differ significantly between the two groups.

## Introduction

Collis (1998) offered a concept for pedagogical re-engineering of existing courses that is more flexible, involves more student engagement and better structure, and is more attuned to students’ responsibility for their own continued learning. Wright and Cordeaux (1996) similarly recommended a shift from instructor-transmission models to learner-oriented classrooms, taught according to the process-based model, to achieve effective learning. Innovative pedagogy can provide opportunities to help the teacher and his/her students develop their identities in relation to each other. With the support of information and communications technology (ICT), which plays an increasingly important role in education for sustainable development (Carrión-Martínez et al., 2020), learners in an innovative pedagogy classroom take responsibility for communicating their meanings that freely convey socio-affective and meta-cognitive factors through social interaction (Farren, 2016). In line with these ideas, many innovative pedagogies have been proposed, for example, inquiry-based learning (Schwab, 1962), problem-based learning (Servant-Miklo, 2019; PBL was first implemented in 1969 at McMaster University School of Medicine), play-based learning (Cheng and Stimpson, 2004), and design-based learning (Nelson, 1984).

Based on concepts explored during the 1990s, Baker (2000) presented the pedagogy of flipped classroom, which captures the essence of innovative pedagogy. With the support of mobile technology, such as phones and tablets, which can afford new opportunities to directly influence learning processes and outcomes (Bernacki et al., 2020), the flipped classroom is being implemented in a wide range of disciplines (mathematics, social sciences, humanities, etc.) at a variety of educational levels across many countries (Hao, 2016; Hinojolucena et al., 2018; Builfabrega et al., 2019; Su et al., 2019a,b, 2020). Many instructors in traditional higher education institutions have therefore considered the need to redesign their instruction to flipped classes. For example, Lombardini et al. (2018) designed two flipped classrooms, partial flip and full flip, to examine students’ learning effectiveness in a microeconomics course, and found that the students were not as satisfied with full flip as with partial flip due to the workload. Many studies have combined the task-driven instructional approach with the flipped classroom and evaluation shows that it has a certain degree of success in its application (Yin, 2015; Li, 2017; Hua, 2019; Su and Wu, 2021).

The term “playful learning” was defined by Kangas et al. (2017) as learning activities that are designed and implemented to promote students’ playful engagement and exploration. Playful learning has also been shown to facilitate students’ creativity and imagination (Kangas, 2010a). Offering an active learning method, PLE aims to physically engage students in learning tasks (De Koning-Veenstra et al., 2014). In the PLE, novel tools and technologies can be applied to support learning. Students can learn with imagination and a playful attitude. However, the studies that have reviewed students’ learning engagement and learning outcomes in flipped classroom hands-on making courses in a PLE are few. In a PLE, learning occurs through various playful and physical learning activities, including hands-on and body-on activities (Säljö, 2005, 2006). Due to the content of hands-on making courses being more related to practical knowledge, students can benefit from a PLE, thus anchoring their knowledge (Rajesh et al., 2019). Therefore, this study took a hands-on course, *Media Making with Chinese Culture*, to explore participants’ learning effectiveness with the flipped classroom approach in a PLE.

Empirical studies on learning effectiveness usually measure improvement on only one aspect of learning performance or a single learning outcome. An evaluation framework that measures the overall learning performance or learning outcome should be designed to fully describe the effectiveness of a flipped classroom approach. According to such a framework, each learning performance dimension can be compared to identify which learning aspects have been improved in a given flipped classroom; for instance, collaborative learning strategies’ effect on learner performance (McDonough and Foote, 2015). Few studies have integrated the different dimensions of effectiveness into an evaluation framework that allows for comparisons of change across dimensions. To fill this research gap, this study provided a detailed description with empirically task-driven instructional adjustments during three rounds of pilot testing, which was customized and applied to a conventional classroom to promote students’ learning effectiveness. The study further proposed an evaluation framework for comparing the learning performance of the two groups of students so as to determine the effectiveness of the flipped classroom approach in a PLE.

## Literature Review

### Flipped Classroom

The flipped classroom was first defined in 1996 (Lage et al., 2000) as an “inverted classroom” that involved a significant change in the order of pre-class and in-class instruction. Baker (2000) suggested the name “classroom flipping” consistent with the changing role of teachers. Teachers changed from being the “Sage on the Stage” to the “Guide on the Side” in the flipped classroom, and the content to be learned was not taught by the teacher via face-to-face classroom interactions but was instead learned by the students themselves outside the classroom via online learning using various resources. In the conventional teacher-centered classroom instruction model, teachers instruct students in the classroom. Compared with this traditional model, the flipped classroom instructional model offers new possibilities in terms of incorporating digital instructional materials to increase teachers’ ability to teach concepts, motivate students to learn, and promote learning achievements (Kazanidis et al., 2019).

Many pedagogies have been implemented in flipped classroom settings, for example, task-oriented project-based teaching (Li and Ma, 2015), problem-based teaching (Feng et al., 2016), inquiry-based learning (Kim and Ahn, 2017), game-based learning (Cheng et al., 2018), and project-based learning (Fan, 2018). These new models have the potential to enhance students’ participation in the learning environment, improve the learning process, and advance performance results (Yilmaz, 2016).

### Design-Based Research

Design-based research (DBR), also referred to as design research, design experiment, and development research (van den Akker, 1999), is a theoretical framework focused on real-world problems with the goal of improving learning. The DBR output contributes with both theoretical knowledge and societal education. Reeves (2006) summarized the DBR in four phases: (1) analyzing the problems from the authentic classroom, (2) designing and developing solutions according to students’ prior knowledge, (3) evaluating the effectiveness of the solutions in the authentic classroom, and (4) providing insights into the whole design process and its principles. Although few studies have reported on the entire DBR procedure, a review revealed that this approach has offered promising results (Anderson and Shattuck, 2012). In Vanderhoven et al.’s (2016) study, the DBR approach has been used to develop effective educational materials to teach children in secondary education (aged 12–19 years) how to act safely on social network site and indicated that DBR approach effectively led to practical solutions and design principles.

### Task-Driven Instructional Approach in Flipped Classroom

In the task-driven method, the teacher arranges tasks for students to complete autonomously to achieve knowledge construction (Sun and Wu, 2018). Based on the task-driven method, students perform learning activities on their own and, thus, gradually cultivate their capacity for independent exploration and autonomous learning (Liu and Niu, 2018). The task-driven instructional approach has been applied in subjects such as English, Computer studies, and Engineering. Liu and Niu (2018) showed that the task-driven method has a great advantage over traditional teaching methods. In recent years, that research has emphasized on task-driven instructional approach in the flipped classroom. Kakh and Mansor (2014) applied task instruction in graduate students’ writing instruction. They proposed that task preparation should consist of selecting appropriate themes of the task, designing task activities, validating the task, piloting the task, and engaging the participants in doing the task. Yin (2015) designed a task-driven model in a flipped classroom and the practice showed that it had a certain degree of success in its application. Li (2017) designed a task-driven model based on the flipped classroom for a “database principles” course, while Hua (2019) constructed a task-driven teaching mode based on the concept of the flipped classroom and found that it could better exert the advantages of flipped classrooms and as a result enhanced the teaching effect. Thus, the present study designed and implemented a task-driven instructional approach in a flipped classroom (TDIAFC) for an undergraduate hands-on making course.

### Evaluation Framework of Learning Effectiveness

Learning outcomes are the knowledge or skills students have acquired by the end of an instructional period. Bloom’s taxonomy (Bloom et al., 1956) described three domains of learner achievement: cognitive, affective, and psychomotor domains (see Table 1). The cognitive domain is the requirement of knowledge and mental skills (Wilson, 2017). In Shi’s research (Shi et al., 2020), college students’ cognitive learning outcomes in the flipped classroom were analyzed. In contrast to the cognitive domain, the affective domain refers to attitudes, emotion, and feelings. Recently, the affective domain has received increasing attention, and it has been researched in several fields, including science (Jeong et al., 2019) and medicine (Pagatpatan, 2020). The psychomotor domain was firstly described in 1964 (Krathwohl et al., 1964) and is composed of utilizing and coordinating motor skills. Sicherl-Kafol et al. (2014) stated that, in comparison to the cognitive and affective domains, little work had been done in the psychomotor domain. However, more research has been conducted on this domain in recent years. In previous studies, the learning performance had been evaluated according to many separate dimensions, but few studies integrate the different dimensions of effectiveness into an evaluation framework.

**TABLE 1:** Aspects of Bloom’s taxonomy domains.

**Domain**	**Aspects**
Cognitive	Intellectual capability, shown as knowledge or “think.”
Affective	Feelings and emotions, shown as attitude or “feel.”
Psychomotor	Manual and physical skills, shown as skills or “do.”

According to Rahmat and Saudi (2008), each domain can be divided into three levels, respectively, describing the content of thinking, feeling, and doing (see Table 1). All three domains have been considered in evaluations of learning performance (Handayani et al., 2018; Günes̨ and Y1lmaz, 2019; Su and Chen, 2020). The learning performance evaluation framework in this study referred to all the three domains.

### Playful Learning Environment

Kangas (2010a) proposed the playful learning environment (PLE), which is a novel and pedagogically validated learning environment that included both the indoor and outdoor learning environment with ICTs supported. The features of playful learning are connected with collaboration, playfulness, creativity, narration, emotion, embodiment, and media richness (Hyvonen, 2008; Kangas, 2010a,b). For learners, playfulness is an attitude to learning through play or games in a PLE (Säljö, 2005, 2006). Existing studies showed learning in a PLE had positive effects on academic achievements for school students and learners in working life (Sawyer, 2006). Kangas et al. (2017) proposed that playful learning was an effective learning approach with ICTs tools constructing a PLE, because learners in PLE showed an active playful attitude and full of imagination through the learning process. Studies also supported the idea that PLE could promote engaging, insightful, and hands-on learning that usually produced a joy of learning (Csikszentmihalyi, 1990; Resnick, 2006; Kangas, 2010b).

However, although many studies support the learning approach in a PLE to promote learners’ learning, the learners cannot automatically benefit from the learning process. Johnson (2004) introduced the play evaluation continuum to illustrate the influence of the playful learning approach on learners. There are both positive and negative effects of playing learning on learners. Playing or a game may be psychologically harmful to a learner, other learners, or the learning environment. Therefore, it is significant to make rules for play and create evaluation tools to evaluate the effectiveness of the learning approach in a PLE. In the study, PLE was constructed in the flipped classroom approach to promote learners learning. With the combination of task-driven and evaluate the framework of learning effectiveness, it was expected that the positive effects of both the flipped classroom and the PLE can bring benefit to learners.

### Research Hypotheses

The aim of the study was to design a model that embeds features of Chinese culture in media making. TDIAFC was implemented in this course.

In the flipped classroom, rather than using the class time to transmit knowledge to the students by lecturing, the teacher engages with the students via discussion, solving problems, hands-on activities, and scaffolding (Akçay1r and Akçay1r, 2018). For the purpose of analyzing how this flipped strategy influences students’ learning effectiveness, the following research hypotheses are proposed:

H1: Students’ learning effectiveness is significantly improved under the revision of the task-driven instructional approach in the three-round flipped classroom, which provides a personalized design of the flipped classroom approach.

H2: According to the evaluation framework, students’ learning performance differs significantly in three domains (i.e., cognitive, affective, and psychomotor) between students who participate in flipped learning instruction and their peers who participate in lecture-centered classroom instruction.

## Materials and Methods

### Participants

Participants comprised two groups of students (see Table 2) who enrolled in a hands-on course named *Media Making with Chinese Culture* at a university. The course, among the most popular general education elective courses at this university, focuses on using ICTs and mobile technology to convey the meanings of Chinese traditional culture. The course is also an open online course on the *iCourse* platform. All study participants were sophomores or juniors, as the course is not open to first-year students; all freshmen are required to pass a computer test at the end of their first year to ensure that they have obtained the basic ICTs and mobile technology skills to study online. The course is an open general education elective course for any interested student of any major. Considering the capacity of the classroom for the face-to-face instruction, about 80 students consisted of one class section. The course was so popular that 160 students enrolled in the same semester. While 81 students enrolled in the course as one class section, another class section was open for the rest 79 students who wanted to enroll in the course. Finally, there were two class sections of 81 and 79 students enrolling in the course in the same semester. Thus, the two class sections formed the EG of 81 participants and the CG of 79 participants. Participants were randomly assigned to the two groups.

**TABLE 2:** Gender and majors of the two groups.

		**Gender**		**Major**		
	**N**	**Male**	**Female**	**Humanities, arts,**	**Natural**	**Teacher education**
				**and social sciences**	**science**	**and other majors**
EG	81	38%	62%	66%	26%	8%
CG	79	44%	56%	51%	25%	24%

*EG, experimental group; CG, control group. In Chinese normal universities, the number of female undergraduates is usually greater than the number of male students.*

In this study, both the EG and CG students enrolling in the hands-on course were instructed in the same PLE that was equipped with multiple technologies associated with rich media tools for students to create their hands-on works. There were enough computers installed with various software and available access to online tools for each student to design and make the works. For example, KAHOOT, a web-based platform that allows users to easily create and play an interactive, multiple-choice-style game (Zucker and Fisch, 2019), was provided for playful learning in the classroom. Other equipment, such as cameras, video cameras, three-dimensional (3D) printers, and other hands-on making materials, were also provided in the classroom. Additionally, learning resources such as lectures, tutorials, and training videos pertaining to Chinese culture for knowledge learning and skills training were also provided. With the tools, technologies, and materials, a playful and engaging learning environment was developed. In the PLE, students learning through playfulness can design and create their hands-on works about Chinese culture.

### Instruments

Students’ learning effectiveness was evaluated by the pre-test score, summative score, final score, and learning performance evaluation. The pre-test score was the score of the first coursework assignment. The summative score was the calculation of the last four coursework assignments. The final score calculating the scores of five coursework assignments, was the comprehensive score for each student as the course score. All tests were calculated by a 100-point scale, which was also the requirement for the course grade at the university. Finally, all the results of the tests for the two groups would be compared.

The learning performance was evaluated by the learning evaluation framework designed as a questionnaire based on Bloom’s taxonomy of domains. This framework covered the cognitive, psychomotor, and affective domains. Six variables were defined according to the three domains. The first variable, knowledge mastery and skills application (KNO), was based on the cognitive domain and “deal[t] with the recall or recognition of knowledge and the development of intellectual abilities and skills” (Bloom et al., 1956, p. 7). In Bloom’s taxonomy, the psychomotor domain was not mentioned in detail (Bloom et al., 1956, pp. 7–8). The second through fifth variables in the psychomotor domain, based on the instructional design and learning requirement of the hands-on course and previous studies (Edward, 2002; Krivickas and Krivickas, 2007), were, respectively, defined as self-learning (SELF), the utilization of learning resources (UTI), collaborative learning (COL), and expression and communication (EXP). The sixth variable, attitude and affectivity (ATT; Syaiful et al., 2019), was based on the affective domain and “describe[s] changes in interest, attitudes, and values, and the development of appreciations and adequate adjustment” (Bloom et al., 1956, p. 7).

The questionnaire (see Appendix) mainly comprised three sections corresponding to the three domains. The cognitive domain included three items for the variable of KNO. The items relating to the psychomotor domain consisted of four items for SELF, three items for UTI, five items for COL, and four items for EXP. Finally, five items related to the affective domain for ATT. A Likert-type scale from 1 (strongly disagree) to 5 (strongly agree) was applied to each item in the three domains.

### Design of the Study of Three-Round TDIAFC

**FIGURE 1:**
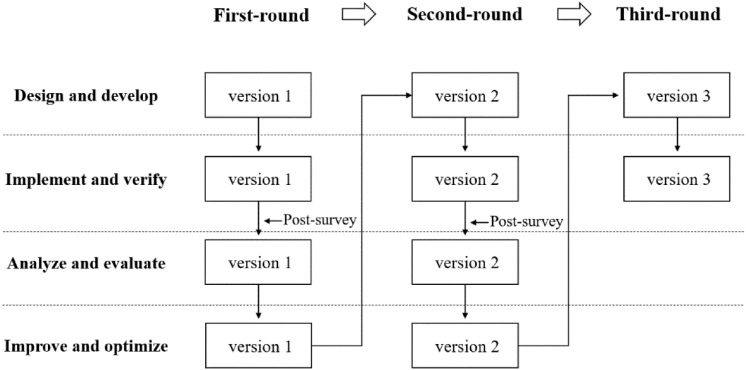
Design of TDIAFC.

According to the design-based and the task-driven research approach, this study designed four interconnected phases: (1) design and develop TDIAFC, (2) implement and verify TDIAFC, (3) analyze and evaluate TDIAFC, and (4) improve and optimize TDIAFC. Vanderhoven et al. (2016) stated that DBR needed multiple iterations. In this study, a three-round experiment of TDIAFC was carried out (see Figure 1).

In the first round, practitioners and teachers designed version 1 of TDIAFC based on the theory of constructivism. After implementing version 1 of the experiment, a first post-experiment survey was designed to identify the problems in the first round. Based on the feedback on the first round of the study, version 1 of TDIAFC was revised and version 2 was developed. In the second round, version 2 of TDIAFC was implemented. The same post-experiment survey as used in the first round was administered to identify the problems in the second-round experiment. Based on the feedback of the second round of the study, version 2 of TDIAFC was revised and version 3 was developed. In the last round, version 3 was implemented. Version 3 (see Table 3) includes learning tasks that were specifically designed from the beginning to the end of the flipped classroom instruction.

**TABLE 3:** Teacher and students’ tasks of version 3 of TDIAFC.

	**Before-class**	**In-class**	**After-class**
Teacher tasks	1. Confirm learning objectives and make a concept map. 2. Prepare the learning content and other learning resources to be shown on a concept map. 3. Design several before-class tasks. 4. Send all learning materials to the online platform. 5. Summarize all questions students pose before class in the online platform. 6. Design in-class learning activities and predict the time needed to carry them out. 7. Design rubrics for formative and summative evaluations.	1. Provide instruction for crucial points and problems that students do not understand and cannot solve. 2. Use Kahoot to test students’ mastery of pre-class learning outcomes. 3. Guide the learning activity. 4. Facilitate student presentations and control time limits. 5. Give comments to students’ learning task individually and in groups. 6. Summarize students’ performance for individuals and groups. 7. Evaluate the performance using the rule of bonus points.	1. Conduct the post-test for learning outcomes. 2. Evaluate the performance of each student and group before and during class. 3. Evaluate learning tasks (i.e., student presentations). 4. Summarize the evaluation and announce evaluation results. 5. Reflect on the flipped classroom model as a whole.
Student tasks	1. Make sure all learning materials are received. 2. Study learning materials at their own pace in groups. 3. Complete the learning task and prepare the class presentation, dividing tasks and cooperating. 4. Finish the test of pre-class learning content on “Questionnaire Star.” 5. Send problems to the online platform.	1. Listen to teacher instructions until they understand and resolve the problems they posed before class. 2. Join in the learning activity. 3. Present group learning outcomes according to the pre-class learning task. 4. Evaluate their own and other groups’ presentations according to teacher-designed rubrics.	1. Complete the online post-test and survey. 2. Revise presentations according to advice from the teacher and other classmates. 3. Upload final learning assignments to the online platform. 4. Check the evaluation result.

### Procedure

Both the EG and the CG students experienced 8 weeks of learning, which covered five themes. The EG students were instructed applying TDIAFC, and they experienced a three-round instructional process. In the 1st week, the EG received basic readiness training; they then experienced the first round of version 1 of TDIAFC in the 2nd and 3rd week to complete the pre-class, in-class, and after-class tasks for the first theme. In the second round of flipped classroom instruction, the EG participants were instructed by version 2 of TDIAFC in the 4th and 5th week to complete the second and third themes. In the third round, they were instructed using version 3 of TDIAFC in the 6th and 7th week to complete the last two themes. Thus, there were five themes and five coursework assignments for both groups.

The teacher, learning content, and coursework were the same for the CG and the EG participants, but the instruction was presented for the former via lectures and without any of the components of the flipped approach. The course adopted the group cooperative learning approach, which was consistent with peer instruction theory (Crouch and Mazur, 2001). Peer instruction makes use of student interaction and helps students to increase engagement, understanding, and problem-solving ability. For each lesson, the students of each class section completed the designed tasks in groups. In the final week, the evaluation of all coursework was summarized. The questionnaire, which was sent to an online survey tool named *Questionnaire Star*, was open to the two groups as well. Before submitting their response, the participants were informed that they were participating in this study. The study aimed to promote instructional strategies and the findings of the study would be published. The data they provided was anonymous and would not be of any commercial use or influence their final course scores. All the students agreed to participate in the study.

## Results

### Students’ Summative and Final Score

From the results (see Table 4), it was found that the EG students’ mean scores were higher than those of the CG students. However, the value of the standard deviation of the final score of the EG was less than that of the CG. This indicates that the dispersion degree of the scores of students in the EG was small. Students’ scores in the CG varied more widely.

**TABLE 4:** Score *t* test for the EG and CG students.

**Score**	**EG (*N* = 81)**		**CG (*N* = 79)**		***t* test score**	**Effect size (Cohen’s d)**
	**M**	**SD**	**M**	**SD**		
Pre-test (1st coursework)	81.51	5.278	79.53	15.274	0.280	0.173
2nd coursework	81.64	5.168	81.19	5.994		
3rd coursework	83.81	4.204	82.49	5.084		
4th coursework	85.49	5.218	83.15	5.388		
5th coursework	95.12	7.825	94.68	7.899		
Summative	84.85	2.900	83.58	4.333	0.032*	0.344
Final	84.62	2.893	83.29	4.813	0.035*	0.335

CG, control group; EG, experimental group; M, mean; SD, standard deviation. *0.01 < *p* < 0.05 shows difference.

In this study, *t* testing and the effect size were analyzed to check whether there was a difference between the two groups. The score of the first coursework seemed to be the score of the pre-test. The result of the *t* test for the pre-test showed that there was no significant difference between the two groups (*p* > 0.05). The study also analyzed the scores of the summative and final score. The result of the *t* test of the summative score showed that there was a significant difference between the two groups (0.01 < *p* < 0.05). There was also a significant difference in the final score between the two groups (0.01 < *p* < 0.05). Cohen’s *d* is the most commonly used standardization effect in the *t* test (Cuthill et al., 2007) and is defined as the difference between the mean values of two groups divided by the standard deviation (equation 1). It can be applied to the calculation of effects by comparing the mean values of two groups of samples (Sullivan and Feinn, 2012). The evaluation criteria of Cohen’s *d* are as follows: small effects (≥0.2 and <0.5); moderate effect (≥0.5 and <0.8); large effect (≥0.8) (Cohen, 1988). The values of effective size of the summative and final scores were 0.344 and 0.335, which showed small effects, indicating that the flipped classroom approach had an impact on students’ academic performance.

### Students’ Learning Performance Evaluations

Students’ learning performance was evaluated by a questionnaire based on the evaluation framework. Since the questionnaire was designed by the authors, the reliability and validity of the questionnaire must be tested. Thus, exploratory factor analysis and confirmatory factor analysis were conducted on the questionnaire. The results of exploratory factor analysis are shown in Tables 5, 6, which filter items and define item dimensions. The confirmatory factor analysis results are shown in Tables 7, 8. Table 7 proved that the questionnaire had good aggregation validity, and Table 8 proved that the questionnaire had good discriminative validity. In a word, this questionnaire was effective and reliable and could be used to evaluate the difference between the two groups.

**TABLE 5:** The characteristic values and contribution rates of the six factors in the model.

**Component**	**Eigenvalue**	**Percentage of**	**Cumulative variance**
		**variance**	**contribution rate**
1	5.016	20.902	20.902
2	4.216	17.566	38.468
3	3.479	14.497	52.965
4	2.833	11.804	64.769
5	1.892	7.883	72.652
6	1.244	5.182	77.834

**TABLE 6:** Factor loading of each item in the six-factor model.

	**Item**	**Factor 1**	**Factor 2**	**Factor 3**	**Factor 4**	**Factor 5**	**Factor 6**
Collaborative learning (5 items)	i13	0.889					
	i15	0.884					
	i12	0.873					
	i14	0.855					
	i11	0.816					
Attitude and affectivity (5 items)	i24		0.918				
	i22		0.886				
	i23		0.857				
	i21		0.804				
	i20		0.775				
Expression and communication (4 items)	i19			0.943			
	i17			0.919			
	i18			0.913			
	i16			0.895			
Self-learning (4 items)	i5				0.880		
	i6				0.841		
	i4				0.812		
	i7				0.805		
Knowledge mastery and skill application (3 items)	i3					0.897	
	i2					0.859	
	i1					0.828	
Utilization of learning resources (3 items)	i9						0.860
	i8						0.807
	i10						0.747

*Extraction method: principal component analysis; Rotation method: Caesar normalizing maximum variance method.*

**TABLE 7:** Results of confirmatory factor analysis (*n* = 160).

**Latent Variable**	**Measure item**	**Standardized Factor Loading**	**Composite reliability(CR)**	**Average variance extracted (AVE)**	**Cronbach’s α**
KNO (3 items)	KNO1	0.783	0.8553	0.6641	0.853
	KNO2	0.781			
	KNO3	0.877			
SELF (4 items)	SELF1	0.854	0.8935	0.6775	0.891
	SELF2	0.841			
	SELF3	0.822			
	SELF4	0.773			
UTI (3 items)	UTI1	0.739	0.828	0.6169	0.822
	UTI2	0.850			
	UTI3	0.763			
COL (5 items)	COL1	0.742	0.9186	0.6936	0.916
	COL2	0.842			
	COL3	0.877			
	COL4	0.824			
	COL5	0.872			
EXP (4 items)	EXP1	0.911	0.9453	0.812	0.945
	EXP2	0.889			
	EXP3	0.919			
	EXP4	0.885			
ATT (5 items)	ATT1	0.703	0.9077	0.665	0.906
	ATT2	0.743			
	ATT3	0.846			
	ATT4	0.839			
	ATT5	0.927			

**TABLE 8:** Correlation coefficient matrix and square roots of AVE (*n* = 160).

**Construct**	**KNO**	**SELF**	**UTI**	**COL**	**EXP**	**ATT**
KNO	**0.815**					
SELF	0.297**	**0.823**				
UTI	0.259**	0.532**	**0.785**			
COL	0.110	0.088	0.082	**0.833**		
EXP	–0.19	0.044	0.014	0.145	**0.901**	
ATT	–0.009	0.039	0.065	0.060	0.189*	**0.815**

*0.01 < *p* < 0.05 shows difference; **p < 0.01 shows the obvious difference.

SPSS 24.0 was used to conduct exploratory factor analysis on the scale and rotated the factors with the maximum variance method. The Cronbach’s alpha coefficient was used to test the reliability of the questionnaire. Cronbach’s alpha for the total questionnaire was 0.813, indicating that the questionnaire was highly reliable. The KMO value in this study is 0.790 (higher than 0.7), and the results of Bartlett’s test of sphericity showed correlations among the different variables (χ^2^ = 2756.482, *p* = 0.000 <0.001), indicating that the data are suitable for EFA.

To measure the validity of the dimension, the study used the principal component extraction method to extract the factor and obtain six factors; the items of each variable were on the same dimension, indicating that the questionnaire dimension was effective (Conway and Huffcutt, 2016). To determine the interpretability of factors, the study used the maximum variance rotation method to rotate and obtain the component transformation matrix, as shown in Table 5. The factor load of each factor was greater than 0.5, proving that each factor had good interpretability (Fabrigar et al., 1999).

In this study, the principal component analysis was used to extract factors, and exploratory factor analysis was conducted with the maximum variance rotation method. Factors with an eigenvalue greater than 1 were selected. After multiple orthogonal rotations, the items with factor loads less than 0.4 and inconsistent contents were deleted. Finally, 24 items with eigenvalues greater than 1 and an independent factor load greater than 0.5 were obtained (Fabrigar et al., 1999). Six factors were extracted, and the cumulative variance contribution rate was 77.834% (Conway and Huffcutt, 2016). The eigenvalue, variance contribution rate, and cumulative variance contribution rate of the six factors are shown in Table 5.

The factor load after rotation was shown in Table 6. Factor 1 (knowledge mastery and skill application, KNO) contained three items – i1, i2, and i3 – explaining the total variation of 20.902%. Factor 2 (self-learning, SELF) contained four items – i4, i5, i6, and i7 – that could explain 17.566% of the total variation. Factor 3 (utilization of learning resources, UTI) contained three items – i8, i9, and i10 – which accounted for 14.497% of the total variation. Factor 4 (collaborative learning, COL) contained five items – i11, i12, i13, i14, and i15 – that accounted for 11.804% of the total variation. Factor 5 (expression and communication, EXP) contained four items – i16, i17, i18, and i19 – that accounted for 7.883% of the total variation. Factor 6 (attitude and affectivity, ATT) contained five items – i20, i21, i22, i23, and i24 – that accounted for 5.182% of the total variation.

Table 7 showed the reliability and validity of each dimension’s items, with each dimension showing acceptable internal consistency (Cronbach’s alpha ranging from 0.822 to 0.945), which was adequate for the factor analysis (Bagozzi and Yi, 1988). When the AVE of all factors of the model is greater than 0.5, the convergence validity of potential variables is better (Fornell and Larcker, 1981). The composite reliability value was greater than 0.6 and slightly greater than Cronbach’s alpha, so the collected data were very reliable.

Discriminant validity was evaluated by comparing a construct’s square root of AVE with the construct’s correlation coefficient (Fornell and Larcker, 1981). As displayed in Table 8, the values of the square root of AVE of all constructs (marked as bold) were greater than the correlation coefficients, indicating that the discriminant validity between constructs was acceptable and the measurement model had good discriminant validity (Schumacker and Lomax, 2016).

Table 9 shows that the value of EG students’ learning performance had higher mean scores than that of the CG students in each dimension. The difference among the CG students was greater among the EG students in all dimensions from the value of standard deviation. Further, the learning performance gap between students with higher scores than lower ones was more significant in the CG students than in the EG students. Moreover, the EG students performed better than the CG students according to the mean scores, indicating that the students in the EG adapted well to the flipped learning method. The *t* test results showed that there was a significant difference between the two groups in each sub-dimension of learning performance, particularly in the psychomotor domain of collaborative learning.

**TABLE 9:** Results for the evaluation framework.

**Sub-dimension**	**EG (*N* = 81)**		**CG (*N* = 79)**		***t* test**
	**M**	**SD**	**M**	**SD**	
KNO (3 items)	4.06	1.54	3.83	2.61	0.042*
SELF(4 items)	4.03	2.58	3.77	3.63	0.037*
UTI (3 items)	3.66	2.29	3.30	3.08	0.013*
COL (5 items)	3.79	3.76	3.36	5.57	0.005**
EXP (4 items)	3.90	4.31	3.47	5.18	0.023*
ATT (5 items)	3.77	3.93	3.41	5.06	0.013*

*0.01 < *p* < 0.05 shows difference; **p < 0.01 shows the obvious difference; CG, control group; EG, experimental group; M, mean; SD, standard deviation.

## Conclusion

### Problems and Redesign of Three Rounds of the Flipped Classroom Study

Based on the survey results on the first-round study of the EG students, the following three problems were exposed. First, according to the results, 95.13% of the EG said that they had achieved high scores. However, the score for each student could not be obtained. Second, 68.21% of the EG reported that they had spent 2 to 3 h on pre-class self-learning; however, the time each student spent on pre-class learning could not be verified. Third, in-class time was not managed effectively. There was little time for in-class learning activities owing to more time spent on the discussion and answering the questions students posed in the pre-class learning.

In the second round of the study, several changes were made. First, learning concept maps were provided to better align the learning objectives with the learning activities and assessments. Previous research has also shown that concept maps can help learners activate their previous knowledge and establish connections between concepts in later learning (Gurlitt and Renkl, 2009; Schroeder et al., 2017). With the help of concept maps, students could quickly and easily get the key points of the learning requirements in their pre-class learning. Students posed noticeably fewer questions than in the last round. Second, the pre-class test was created and published in *Questionnaire Star*. Students completed the test on the platform of *Questionnaire Star.* Each student’s score could be calculated and identified through the platform. Third, a time limit was set for in-class activities including the group presentations, discussions, and self-evaluations to ensure that they could be completed in time. Version 2 of TDIAFC was developed based on the results of the first round of instruction.

The second round of the experiment resolved the problems of the first round. As a result, the pre-test was uploaded to *Questionnaire Star*, and each student’s pre-class test score could be seen. According to the new results, nearly 70% of the students got the correct answers for approximately 90% of the questions. Time spent on pre-class self-learning was less than in the previous round. Nearly 90% of students said they spent 30–50 min on pre-class learning.

However, other problems appeared. One problem is that in the in-class presentation section, students apparently only listened carefully to their own group presentations. However, they could not focus on the other groups’ presentations. Second, the problem was still in the presentation section; moreover, each group presentation lasted too long to leave sufficient time for the other in-class learning activities.

In the third round of the study, several changes were made. First, bonus points were given to students to motivate their participation in the discussion. Second, the discussion board was used to post each group’s revised presentation. Students were asked to give comments to each group presentation. Students could learn from each other through the online board posts. Version 3 of TDIAFC was designed based on the evaluation of the second round of instruction.

According to the survey of the three-round study, it was found that the coursework score for each group increased from the first to the last unit (see Table 4). Additionally, students paid more attention to other groups’ presentations and participated more actively in the learning activities than before with the new play rules. Moreover, the time of in-class learning activities was under better control.

The EG students’ performance improved significantly throughout the three-round study. The results showed that an increasing number of students adapted to the new instructional pedagogy. Students seemed to spend less time on pre-class activities (from 2 to 3 h to 30–50 min) and achieve higher scores in the pre-class test (from uncertain to 90% correct rate). During in-class time, the students participated more actively in each of the learning activities, such as discussion and comments. The scores of the five coursework assignments of each group showed significant improvement.

### Revision of the TDIAFC Improved Learning

This study applied three versions of TDIAFC in the same course. The design was revised for the first two rounds of the study in three main ways. First, each EG student’s pre-class achievement score was obtained. Second, learning tools such as concept maps and task decomposition were added to scaffold their learning. Third, motivation strategies were implemented and found to be effective in facilitating students’ active participation. The results showed that the EG students performed steadily and incrementally better as they proceeded through the rounds of the class. Additionally, by experiencing the three-round study, the instructors learnt how to better design the learning task, implement the learning activities, and provide learning scaffolding to help students learn independently (González-Gómez and Jeong, 2019), while the students gained knowledge on how to learn in the flipped classroom by becoming familiar with the process and requirements of this type of transformative pedagogy.

In this study, the three-round experiment showed a full picture of the personalized design of the flipped classroom pedagogy. The course, a hands-on making course, to which no other new innovative pedagogy has been applied, required more active participation and deep cooperation with one another. The sample undergraduates had their own learning habits and were accustomed to the lecture-centered classroom. These factors had an obvious impact on the implementation of the flipped classroom approach. The tasks, learning process, and learning outcomes of the three-round study were personalized and specific, but could also provide guidance more generally for those wishing to design or adapt a course to the flipped classroom model. H1 was supported.

### TDIAFC Facilitated Students’ Learning Effectiveness

The study applied TDIAFC in a PLE and achieved a good result, which was similar to other studies (Yin, 2015; Li, 2017; Hua, 2019), which used task-driven model in a flipped classroom and had a certain degree of success in its application. In the study, the EG students’ assessed learning performance were significantly better than those of the CG students, which was consistent with the study of van Alten et al. (2019). In the study, the EG students’ self-learning ability was significantly better than those of the CG students. Previous experiments have also shown that the flipped classroom strengthened the students’ self-learning ability (Ma et al., 2017). Lai and Hwang (2016) hypothesized that students who lack self-learning capabilities might be disadvantaged in flipped classrooms. Across a wide-reaching synthesis of currently available interdisciplinary research reports, Shi et al. (2020) found that the flipped classroom instruction can positively influence college students’ cognitive and affective achievement. The results of Dinndorf-Hogenson et al. (2019) suggested that the methods used with the flipped classroom pedagogy do not significantly affect student performance on psychomotor skill acquisition. However, in this study, the learning performance evaluation of the three-round study showed that all three dimensions of cognitive, affective, and psychomotor differed significantly between the two groups. H2 was supported. However, the polarization of the sub-dimensions of expression and communication and attitude and affectivity was serious. Therefore, future research should pay attention to abilities of expression and communication and the adaptability of students with different self-learning abilities to flipped learning.

### Limitations and Future Work

There are limitations to this study that leaves scope for future studies to further explore the TDIAFC model. First, both the EG and CG students learned in the same PLE. The evaluation of PLE was combined with the evaluation of the flipped classroom approach in the evaluation framework in the study. Therefore, the evaluation of the effects of PLE was not separately proposed in the study. With the results of the evaluation framework, it was obvious that the new approach in a PLE showed a positive effect on learners. Second, increasing the number of experiments may reveal more problems. The design of TDIAFC may thus be revised again. Third, more personalized evaluation tools should be developed to evaluate the learning effectiveness of the flipped classroom approach. Fourth, this research utilized questionnaires to collect data, which means that common method biases may exist. In addition, according to a survey of the number of courses taken by participants in one semester, 56.1% of the participants had 9–12 courses. TDIAFC with more time spent on pre- and post-class learning may increase students’ coursework load, which can decrease their interest and motivation. Thus, this limitation calls for a wider range of evidence to complement these findings.

## Data Availability Statement

The raw data supporting the conclusions of this article will be made available by the authors, without undue reservation.

## Ethics Statement

Ethical review and approval was not required for the study on Human Participants in accordance with the Local Legislation and Institutional Requirements. Written informed consent from the college students/participants was not required to participate in this study in accordance with the National Legislation and the Institutional Requirements.

## Author Contributions

All authors contributed equally to the conception of the idea, implementing and analyzing the experimental results, and writing the manuscript and read and approved the final manuscript.

## Conflict of Interest

The authors declare that the research was conducted in the absence of any commercial or financial relationships that could be construed as a potential conflict of interest.

## Appendix

**Table:** Questionnaire of Learning performance evaluation.

**Domain**	**Sub-dimension**	**Items**
Cognitive	Knowledge mastery and skills application (3 items)	i1. I can remember and understand the knowledge I learned. i2. I can apply the knowledge to real life. i3. I can utilize the skills I learned to create my work.
Psychomotor	Self-learning (4 items)	i4. I can easily download all the online learning resources or get the learning resources the instructor required on time. i5. I can control the time spent on the pre-class self-learning. i6. I can complete the pre-class learning task. i7. I have my way of self-learning.
	Utilization of learning resources (3 items)	i8. I have learned 80% of the online and in-class learning resources. i9. I can quickly find the key points from all the learning resources. i10. I can easily find the answers to my questions in the learning resources.
	Collaborative learning (5 items)	i11. I am active in the group discussion and express my own opinion. i12. I am in charge of one part of the group coursework. i13. I can carefully listen to others or express my concerns promptly in online group discussions. i14. I can respond to my group members on time and give thought to their suggestions. i15. I gain a lot from group learning.
	Expression and communication (4 items)	i16. I share my opinions with others in the discussion. i17. I gain suggestions for further revision of my coursework from others. i18. I can quickly and precisely express my opinions. i19. Others can understand my point of view.
Affective	Attitude and affectivity (5 items)	i20. The content of the course is interesting. i21. What I learned in the course is of great value. i22. I like the pedagogical strategy the instructor applied in the class. i23. I am fully involved in the class. i24. I will recommend the course to others.
