# Corrigendum: Innovative Pedagogy and Design-Based Research on Flipped Learning in Higher Education

**DOI:** 10.3389/fpsyg.2021.838081

**Published:** 2022-02-03

**Authors:** Li Zhao, Wei He, Yu-Sheng Su

**Affiliations:** ^1^School of Education Science, Nanjing Normal University, Nanjing, China; ^2^Department of Computer Science and Engineering, National Taiwan Ocean University, Keelung, Taiwan

**Keywords:** flipped learning, innovative pedagogy, higher education, playful learning environment, design based research

In the original article, there was an error in the Funding statement as published. The funder name was incorrectly written as “Ministry of Science and Technology, Taiwan, R.O.C.” The corrected Funding statement is shown below.

This research was funded by the National Social Science Foundation of China (Grant No. BCA200093) and Priority Academic Program Development of Jiangsu Higher Education Institutions. In addition, this study was supported by the Ministry of Science and Technology, Taiwan, under grants MOST 109-2511-H-019-004-MY2 and MOST 109-2511-H-019-001.

The authors apologize for this error and state that this does not change the scientific conclusions of the article in any way. The original article has been updated.

## Publisher's Note

All claims expressed in this article are solely those of the authors and do not necessarily represent those of their affiliated organizations, or those of the publisher, the editors and the reviewers. Any product that may be evaluated in this article, or claim that may be made by its manufacturer, is not guaranteed or endorsed by the publisher.

